# Subtractive proteomics and reverse-vaccinology approaches for novel drug targets and designing a chimeric vaccine against *Ruminococcus gnavus* strain RJX1120

**DOI:** 10.3389/fimmu.2025.1555741

**Published:** 2025-04-14

**Authors:** Hou Dingding, Sher Muhammad, Irfan Manzoor, Sana Abdul Ghaffar, Hissah Abdulrahman Alodaini, Nadine MS. Moubayed, Ashraf Atef Hatamleh, Xu Songxiao

**Affiliations:** ^1^ Department of Clinical Laboratory, Zhejiang Cancer Hospital, Hangzhou Institute of Medicine (HIM), Chinese Academy of Sciences, Hangzhou, Zhejiang, China; ^2^ Postgraduate Training Base Alliance of Wenzhou Medical University (Zhejiang Cancer Hospital), Hangzhou, Zhejiang, China; ^3^ Faculty of Agriculture and Veterinary Sciences, Superior University Lahore, Lahore, Pakistan; ^4^ Department of Bioinformatics and Biotechnology, Government College University Faisalabad (GCUF), Faisalabad, Pakistan; ^5^ Department of Botany and Microbiology, College of Science, King Saud University, Riyadh, Saudi Arabia

**Keywords:** *Ruminococcus gnavus*, multi-epitope vaccine, reverse vaccinology, inflammatory bowel disease (IBD), subtractive proteomics, immunoinformatics

## Abstract

*Mediterraneibacter gnavus*, also known as *Ruminococcus gnavus*, is a Gram-positive anaerobic bacterium that resides in the human gut microbiota. Notably, this bacterium plays dual roles in health and disease. On one side it supports nutrient metabolism essential for bodily functions and on the other it contributes to the development of Inflammatory Bowel Disease (IBD) and other gastrointestinal disorders. *R. gnavus* strain RJX1120 is an encapsulated strain and has been linked to develop IBD. Despite the advances made on its role in gut homeostasis, limited information is available on strain-specific virulence factors, metabolic pathways, and regulatory mechanisms. The study of such aspects is crucial to make microbiota-targeted therapy and understand its implications in host health. A multi-epitope vaccine against *R. gnavus* strain RJX1120 was designed using reverse vaccinology-based subtractive proteomics approach. Among the 3,219 proteins identified in the *R. gnavus* strain RJX1120, two critical virulent and antigenic proteins, a Single-stranded DNA-binding protein SSB (A0A2N5PT08) and Cell division ATP-binding protein FtsE (A0A2N5NK05) were screened and identified as potential targets. The predicted B-cell and T-cell epitopes from these proteins were screened for essential immunological properties such as antigenicity, allergenicity, solubility, MHC binding affinity, and toxicity. Epitopes chosen were cross-linked using suitable spacers and an adjuvant to develop a multi-epitope vaccine. Structural refinement of the construct revealed that 95.7% of the amino acid residues were located in favored regions, indicating a high-quality structural model. Molecular docking analysis demonstrated a robust interaction between the vaccine construct and the human Toll-like receptor 4 (TLR4), with a binding energy of −1277.0 kcal/mol. The results of molecular dynamics simulations further confirmed the stability of the vaccine-receptor complex under physiological conditions. *In silico* cloning of the vaccine construct yielded a GC content of 48% and a Codon Adaptation Index (CAI) value of 1.0, indicating optimal expression in the host system. These results indicate the possibility of the designed vaccine construct as a candidate for the prevention of *R. gnavus*-associated diseases. However, experimental validation is required to confirm its immunogenicity and protective efficacy.

## Introduction

*R. gnavus* is a gram-positive anaerobic bacterium that is a key component of the human gut microbiota, playing significant roles in both health and disease (1). This bacterium is currently of interest due to its association with IBD and its ability to produce pro-inflammatory polysaccharides that modulate host immune responses (2). Among these strains, RJX1120 stands out for its role in gut inflammation and distinctive genetic characteristics (3). *R. gnavus* is mostly considered a commensal organism but often becomes a pathobiont in dysbiotic conditions and can be associated with diseases like Crohn’s disease and ulcerative colitis (4). Its interactions with mucosal surfaces, the production of mucin-degrading enzymes, and its ability to produce immunomodulatory metabolites have been implicated in disease pathogenesis (5). The pathogenic potential of *R. gnavus* underscores the understanding of the virulence determinants and the molecular mechanisms (6). Currently, no licensed vaccine exists for *R. gnavus*, despite its association with inflammatory bowel disease (IBD) and other gastrointestinal disorders. This highlights an urgent need for novel vaccine strategies. Traditional vaccine development relies on culturing and isolating antigens, which is labor-intensive and time-consuming. In contrast, computational vaccine design offers a more efficient and targeted approach by identifying immunogenic proteins through reverse vaccinology and subtractive proteomics. Proteomic studies are very relevant for revealing the specializations of RJX1120 to unveil its potential therapeutic goals and thus help in generating preventive mechanisms (7).

*R. gnavus* exhibits significant adaptability and resilience in the human gut, which may contribute to its potential resistance to therapeutic interventions (2). This bacterium is known for its ability to degrade complex carbohydrates and mucins, producing metabolites such as short-chain fatty acids (e.g., propionate) that enhance its competitiveness and survival in the gut microbiota (8). In addition, the capacity to immunomodulate through immunogenic polysaccharides could enable this microbe to evade host immunity and promote long-term persistence in the gut (3). Although direct evidence for the presence of antimicrobial resistance mechanisms in *R. gnavus* is scanty, the effects of horizontal gene transfer of resistance genes are favored by its metabolic versatility (9). Understanding the mechanism of its resistance, such as its biofilm formation ability and possibly resistance-determining factors, is important to define suitable therapeutic strategies against pathogenic strains without disrupting the homeostasis of gut microbiota (10).

Subtractive proteomics represents a strategic approach for identifying pathogen-specific proteins important for survival but missing from the host proteome (11). In comparison, the unique set of proteins characteristic to *R. gnavus* points out the drug target candidates and vaccine targets (12). In subtractive proteomics, the subtraction of homologous proteins in the host background removes the critical proteins involved in pathogenicity in the bacterium but avoids the unwanted effects that would otherwise result in drug and vaccine therapies (11). A bioinformatics-driven approach called reverse vaccinology complements subtractive proteomics, antigenic proteins to be used in vaccines are analyzed by reverse vaccinology (11). Such proteins are likely to be surface-exposed, conserved among strains, and capable of inducing potent immune responses (12). Reverse vaccinology does not require isolation and culture of the pathogen, making vaccine discovery for complex organisms like *R. gnavus* significantly faster. Subtractive proteomics combined with reverse vaccinology offers an integrative approach to designing new vaccines and drugs against *R. gnavus*. The membrane and secreted proteins are the most relevant for vaccine development because these are exposed to the host immune system (13). Cytoplasmic proteins are the most suitable drug targets, as they are often critical to bacterial metabolism and survival (14). Using these approaches, multiepitope vaccine constructs can be designed by selecting epitopes from membrane-bound proteins that are non-allergenic, antigenic, and non-toxic (15). These vaccines can induce targeted immunity to pathogenic strains of *R. gnavus* and maintain the commensal balance of the gut microbiota.

In order to create a targeted vaccination against *R. gnavus* strain RJX1120, this study explores the pathogenic role of this bacteria in inflammatory bowel disease (IBD). The study finds possible vaccine candidates by searching the entire proteome of *R. gnavus* for essential, antigenic, and non-homologous proteins using a mix of subtractive proteomics and reverse vaccinology. Single-stranded DNA-binding protein (SSB) and cell division ATP-binding protein FtsE are two important antigenic proteins that have been discovered as potential targets for vaccine development. B-cell and T-cell epitopes are included in a multi-epitope vaccination construct to produce a potent and defense-enhancing immune response. To assess the construct’s stability, immunogenicity, and possible effectiveness, it is subjected to molecular docking with human Toll-like receptor 4 (TLR4), molecular dynamics simulations, and in silico immune response simulations. Codon optimization also guarantees effective expression in *Escherichia. coli*, which makes subsequent experimental validation easier. This study’s main premise is that the multi-epitope vaccination will produce a strong immune response against *R. gnavus*, possibly acting as a prophylactic against the diseases it causes while maintaining the balance of the gut microbiota. To verify its immunogenicity and protective effectiveness, more *in vitro* and *in vivo* validation is necessary.

## Materials and methods

### Retrieval of proteome

The complete proteome of *R. gnavus* strain RJX1120 (Proteome ID: UP000234812) was retrieved in FASTA format from the UniProt database (16). A BLASTp search was performed against the Database of Essential Genes (DEG) to identify essential proteins in *R. gnavus* by comparing its proteome with known essential proteins. These proteins are vital for the bacterium’s survival, growth, and key biological processes (17). Being integral components, these proteins are therefore crucial for an organism’s survival in a particular environment (18). For greater refinement, Cello tool, which predicts the subcellular localization of proteins, was used to identify membrane-associated proteins from the list of essential proteins (19). Due to their accessibility to the host immune system, these membrane-associated proteins are highly promising targets for vaccine development (20). Then the screened proteins were analyzed for the presence of antigenicity based on a threshold of 0.5 using Vaxijen server because the proteins with high values are known to induce immense immune response upon exposure in the host (21, 22). The TMHMM v-2.0 server was used to predict potential transmembrane helices in the target proteins (23, 24).

### Selection and assessment of CTL epitope

CTL epitopes for the target molecule were predicted using the MHC-I binding tool on the Immune Epitope Database (IEDB) (25). The consensus method was applied in the MHC-I binding tool to predict CTL epitopes. Epitopes with a consensus score of less than 2 were selected for further study (26). Subsequently, the potential immunogenicity of epitopes selected for CTL usage was assessed by the use of the IEDB immunogenicity tool (27). To confirm that the selected epitopes have strong potential to elicit an effective immune response, their antigenicity was assessed using the VaxiJen v2.0 server with a threshold of 0.5 (28). Only those epitopes that were considered antigenic were selected for incorporation in the vaccine construct (15). It is crucial that the vaccine candidate does not induce allergic or toxic reactions. The allergenic potential of the predicted epitopes was evaluated using the AllerTOP v2.0 server, and their toxicity was evaluated using the ToxinPred server (22, 29, 30). This broad approach ensured that safe and immunogenic CTL epitopes were identified for potential inclusion in a vaccine design.

### HTL epitopes selection and analysis

Helper T lymphocytes (HTLs) are essential players in the adaptive immune system, where they orchestrate both cell-mediated and humoral responses against foreign pathogens (31). HTL epitopes from the target protein were identified by using the MHC Class II binding tool available through the Immune Epitope Database (IEDB) (32). The search was confined to 15-mer HTL epitopes for their binding affinity to a wide range of HLA-DR alleles to comprehensively cover the immunological coverage and were chosen for their optimal binding affinity and immunogenic potential (26). All selected epitopes were evaluated for antigenicity, allergenicity, and toxicity using the VaxiJen, AllerTOP v2.0, and ToxinPred servers, respectively (27).

### LBL epitope identification and analysis

Linear B-cell epitopes are sequences of amino acids on the surface of proteins that can be recognized by antibodies (33). They are recognized by either naturally occurring antibodies or receptors on B cells, therefore able to stimulate cellular and humoral immunity (34). Vaccine development involves such epitopes critically because they enhance adaptive immunity through amplification of defence mechanisms in the immune response (35). The ABCPred server was used to predict linear B-cell epitopes from the target protein, with the threshold set at a minimum of 0.5 for prediction (36). After predicting epitopes, their antigenicity, allergenicity, and toxicity were assessed using the VaxiJen v2.0, AllerTOP v2.0, and ToxinPred servers, respectively (22, 27, 30). This way, only immunogenic, safe, and non-toxic epitopes were selected to be included in vaccine design (15).

### Designing of vaccine construct

The multi-epitope vaccine (MEV) construct was designed by appropriately linking B-cell and T-cell epitopes with an adjuvant (37). Adjuvants are crucial for enhancing the immunogenicity of vaccine constructs, eliciting a stronger immune response in recipients (38). In this study, cholera enterotoxin subunit B (Accession No: P01556) was chosen due to its established ability to enhance the immunogenic potential of vaccine constructs (39). For linking components, EAAAK linkers were used to attach the CTL epitopes to the adjuvant, providing structural stability and maintaining the functional integrity of the epitopes. GPGPG and AAY linkers were used to connect CTL and HTL epitopes, allowing their efficient presentation and enhancing their immune responses (40). Bi-lysine (KK) linkers were employed to preserve the specific immunogenic activity of linear B-cell (LBL) epitopes (41). Systematic arrangement of the construction is essential for immunogenic efficiency and structural stability; thus, the MEV construct represents a promising candidate for vaccine development (42).

### Structural analysis

The structural properties of the MEV construct were analyzed using various bioinformatics tools (43). First, the physiochemical characteristics of the construct, such as theoretical isoelectric point (pI), molecular weight (MW), instability index (II), aliphatic index (AI), Grand Average of Hydropathicity (GRAVY), and *in vivo*/*in vitro* half-life, were evaluated using the ProtParam server (44, 45). Immunological efficacy was ensured by checking the antigenic and immunogenic profiles of the MEV construct using the IEDB immunogenicity tools and the Vaxijen v2.0 server (46). Allergenic potential was computed using the AllerTOP v2.0 server to ensure safety for the construct in its potential application in humans (42). The SOPMA tool was used to predict secondary structural features of the MEV construct, assessing the proportions of random coils, alpha-helices, beta-turns, and extended chains (44, 47). These detailed structural analyses allow researchers to understand stability, functionality, and applicability in the development of vaccines (48).

### Refinement, confirmation, and prediction of tertiary structure

The prediction of the tertiary structure of the MEV construct is crucial for evaluating its structural and functional efficacy (37). For this purpose, the 3D structure of the MEV construct was predicted using the Alphafold server, which is a state-of-the-art tool for accurate protein structure modeling (49). The Galaxy Refine server was then used to refine and optimize the 3D structure for better stereochemical quality, and to minimize any structural errors that may occur (50). After refining the structure, the quality of the model was analyzed using the RAMPAGE server, where it evaluates the quality based on Ramachandran plot statistics (51). ERRAT server was used to check for possible errors and to assess the overall quality and reliability of the 3D structure of the MEV construct (52).

### B-cell epitopes screening

The B-cell epitopes of the MEV construct were screened through the ABCPred online server and Ellipro tool within the IEDB-AR v2.22 suite (53, 54). For the prediction of linear epitopes, the amino acid sequence of the MEV was fed into the ABCPred server with a threshold set at 0.5 and an amino acid length fixed at 15 residues (55). For conformational epitope prediction, default parameters were used with Ellipro to analyze the 3D structure of MEV construct (15). The conformational approach would serve as a complement to these studies to identify linear epitopes, while identifying putative regions of immunogenicity in the MEV construct (56).

### Binding analysis of TLR4 receptor with the designed vaccine

The effective recognition of the designed vaccine by the host’s immune system is crucial for initiating a robust immune response (56). Molecular docking studies were carried out to evaluate the designed vaccine’s binding capability with the immune receptors. For assessing the role of these immune receptors in stimulating the antimicrobial and adaptive immune responses, TLR4, MHC Class I and II were selected (57). TLR4 was selected due to its critical role in recognizing bacterial antigens and initiating an innate immune response (58). Protein-protein interactions between the vaccine construct and these receptors were modeled using the ClusPro server, which is a reliable tool for molecular docking (59). The resulting docked complexes were visualized using Chimera, a visualization tool for 3D molecular structures (60). The interactions within the docked complexes were analyzed by using the PDBsum server that provides detailed insights into interface residues and binding interactions (61).

### Molecular dynamic simulation

Molecular dynamics simulations are computational methodologies used to study the dynamic behavior and stability of molecular systems such as protein-protein complexes (62). Interaction of the designed MEV construct with the selected receptor was analyzed by the iMODS server that proves to be a fast and efficient tool for molecular dynamics studies (15, 63). iMODS facilitates the exploration of dynamic properties and transition pathways between molecular entities to gain actionable insights into conformational changes (63). The stability of the docked complexes was evaluated through key parameters, such as the main-chain deformability plot, covariance matrix, eigenvalue analysis, B-factor values, and the elastic network model (64). These analyses provided a detailed understanding of the mechanical and dynamic stability of the protein-protein interaction (64).

### Immune simulation

The immune response to the predicted vaccine construct was evaluated using the C-ImmSim 10.1 server (53). This platform is designed to simulate the interactions of the immune system, focusing on key functional components such as the bone marrow, lymph nodes, and thymus (65). The simulation was performed using the following input parameters: Human Leukocyte Antigen (HLA) alleles (DRB1 0101, B0702, A0101), a random seed (12,345), a simulation volume of 10, one injection, and 100 steps (66). These alleles were selected for their broad population coverage and their relevance in antigen presentation, ensuring a diverse immune response (67). Other parameters were set to their default values to ensure accurate simulation of the immune response. This comprehensive immune simulation helped assess the potential efficacy and immunogenicity of the vaccine construct in a simulated mammalian immune system environment (67).

### Dry lab cloning and codon optimization

Codon usage is species-specific, and the presence of non-adapted codons in a gene sequence can result in suboptimal expression levels in the host organism (68). To enhance gene expression, it is essential to optimize the codon usage to match the host’s translational machinery (69). In this study, the Java Codon Adaptation Tool (JCAT) was utilized for the optimization and reverse translation of the MEV construct (70). During this process, prokaryotic ribosome binding sites, Rho-independent transcription termination signals, and appropriate restriction enzyme cleavage sites were selected to facilitate efficient expression and cloning (69). Subsequently, the optimized vaccine construct was inserted into the pET30a(+) vector using SnapGene software, ensuring a seamless cloning process for subsequent experimental validation (71).

## Result

### Proteome analysis

The complete proteome of the pathogenic strain *R. gnavus* RJX1120 was extracted from the UniProt database (Proteome ID: UP000234812), and a subtractive genomics approach was applied for the identification of potential vaccine targets against infections caused by *R. gnavus*. The total number of proteins in the proteome of the strain was found to be 3,219, and a comprehensive filtering pipeline was applied in order to find key target proteins. Initially, the database DEG identified 848 proteins that are essential in the proteome of a pathogen for survival and proliferation. These essential proteins were screened using BLASTp for non-homologous proteins, thus narrowing them down to 245. These 245 were again screened for localization of their subcellular positions and antigenicity. From there, the further studies have been conducted to further investigate the remaining 15 membrane-associated proteins that would possibly become vaccine candidates. These proteins were screened for their antigenicity, allergenicity, and stability. Of these, seven had the highest antigenicity, were non-allergenic, and very stable. The transmembrane helices of these were further tested. The two best vaccine candidates were the Single-stranded DNA-binding protein and the Cell division ATP-binding protein FtsE. These proteins exhibited high antigenicity, were non-allergenic and stable and lacked transmembrane helices (**Table 1**).

**Table 1:** Comprehensive details regarding the antigenic vaccine protein derived from *Ruminococcus gnavus*.

Accession no	Protein	Antigenicity	Allergenicity	Toxicity
A0A2N5PT08	Single-stranded DNA-binding protein	0.7402	Non -allergen	Non -toxin
A0A2N5NK05	Cell division ATP-binding protein FtsE	0.5909	Non -allergen	Non -toxin

### Epitope selection phase

Cytotoxic T lymphocyte (CTL), helper T lymphocyte (HTL), and linear B lymphocyte (LBL) epitopes of specific antigenic proteins were predicted during the epitope selection phase. Among the forecasted epitopes, the top seven CTL epitopes were selected for vaccine formulation based on their non-toxic, immunogenic, antigenic, and non-allergenic properties (**Table 2**). Similarly, the top four HTL epitopes exhibiting non-allergenicity, antigenicity, immunogenicity, IFN-gamma induction capability, and non-toxicity were identified for vaccine design (**Table 3**). Additionally, the top two LBL epitopes, characterized by their antigenicity, immunogenicity, non-allergenicity, and non-toxicity, were chosen for incorporation into the vaccine construct (**Table 4**).

**Table 2:** Selected CTL epitopes finalized for vaccine construction targeting *Ruminococcus gnavus*.

Epitope	Protein	Allele	Position	Antigenicity	Immunogenicity
NLKRMKHRNIAK	Cell division ATP-binding protein FtsE	HLA-A*03:01	65-76	0.5296	-0.18793
VARYTVAVDRRF	Single-stranded DNA-binding protein	HLA-A*23:01 HLA-A*24:02	27-38	0.5194	0.26418
SVSGRIQTGSYT	Single-stranded DNA-binding protein	HLA-A*26:01 HLA-A*25:01	72-83	1.376	-0.02448
FRQGMRISVSGR	Single-stranded DNA-binding protein	HLA-A*31:01	65-76	0.9521	-0.23656
VNEMNERVITMK	Cell division ATP-binding protein FtsE	HLA-B*18:01	200-211	0.7641	0.09304
KRMKHRNIAKYR	Cell division ATP-binding protein FtsE	HLA-A*31:01 HLA-B*27:05	67-78	0.7392	-0.21613
NEMNERVITMKQ	Cell division ATP-binding protein FtsE	HLA-B*18:01	201-212	0.6243	0.01653

**Table 3:** Finalized HTL epitopes for vaccine construction targeting *Ruminococcus gnavus*.

Epitope	Protein	Allele	Position	Antigenicity	Immunogenicity
FAEKYFRQGMRISVS	Single-stranded DNA-binding protein	HLA-DRB1*15:02	60-74	0.6184	-0.21432
QGMRISVSGRIQTGS	Single-stranded DNA-binding protein	HLA-DRB1*07:03 HLA-DRB1*13:02	67-81	1.3073	-0.0008
RQGMRISVSGRIQTG	Single-stranded DNA-binding protein	HLA-DRB1*07:03 HLA-DRB1*13:02	66-80	1.2518	0.1341
SATAVARYTVAVDRR	Cell division ATP-binding protein FtsE	HLA-DRB1*08:06	23-37	0.6282	0.38099

**Table 4:** Finalized B-cell epitope selected for vaccine construction against *Ruminococcus gnavus*.

Epitope	Protein	Score	Position	Antigenicity	Immunogenicity
TRAANNKAANNKMEDG	Cell division ATP-binding protein FtsE	0.59	227	1.6355	0.38477
TAANPTMEDGNSINGL	Single-stranded DNA-binding protein	0.7	7	1.0945	-0.01349

### Construction of multi epitope vaccine

**Figure 1:**
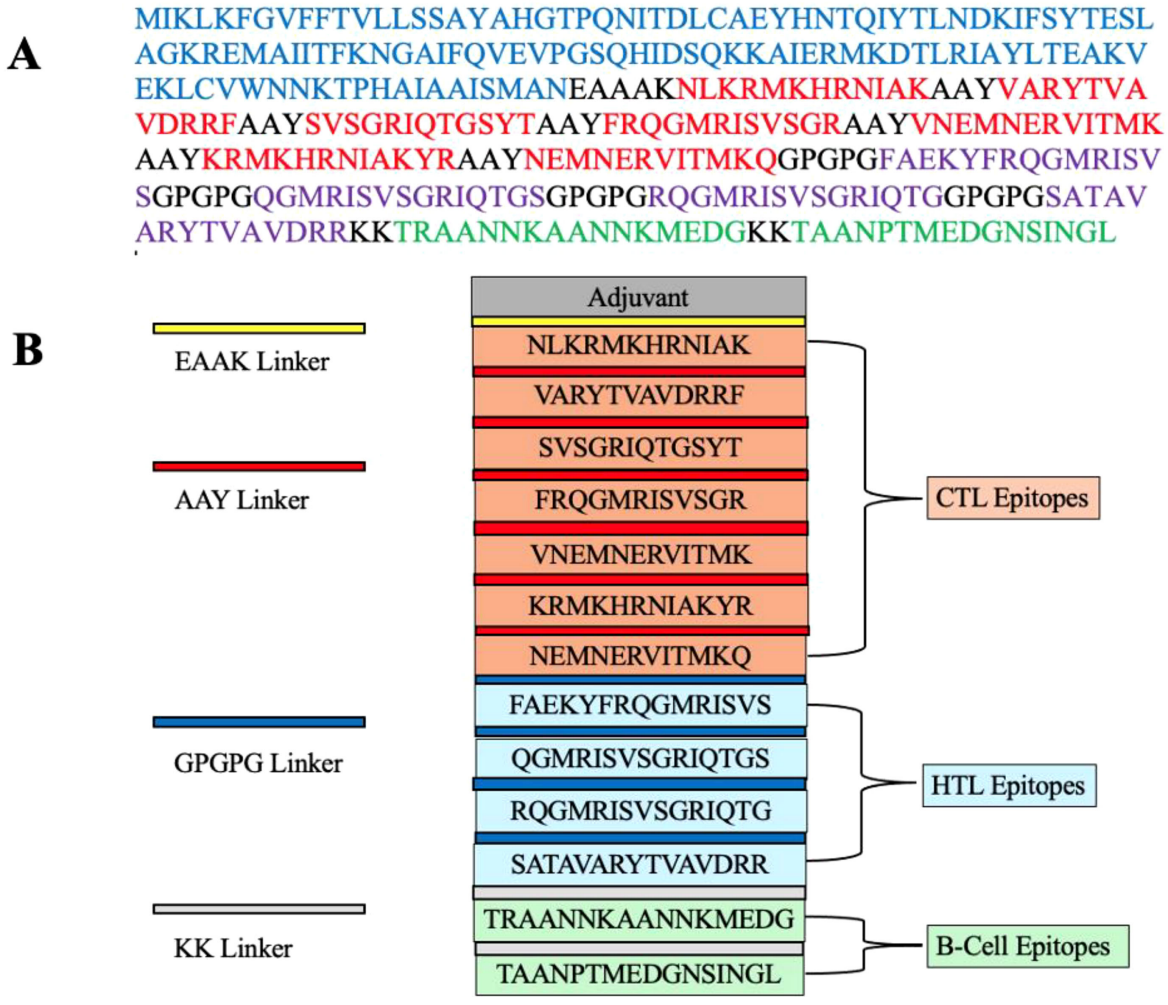
**(A)** A schematic representation of the MEV construct highlights the color-coded elements: the adjuvant (blue), CTL epitopes (red), HTL epitopes (purple), B-cell epitopes (green), and linkers (EAAAK, AAY, GPGPG, KK; all depicted in black). **(B)** The final multi-epitope vaccine (MEV) construct is composed of 347 amino acids. It includes an adjuvant (blue) linked via an EAAAK linker (black) and is connected to CTL epitopes (red) using an AAY linker (black). HTL epitopes (purple) are joined by GPGPG linkers (black), while KK linkers (black) connect B-cell epitopes (green).

The vaccine construct was designed by integrating 7 CTL epitopes, 4 HTL epitopes, and 2 B-cell epitopes with a suitable adjuvant and linkers. Cholera enterotoxin subunit B, consisting of 236 amino acids, was incorporated at the N-terminal of the vaccine using an EAAAK linker to enhance immunogenicity. The CTL, HTL, and B-cell epitopes were linked using AAY, GPGPG, and KK linkers, respectively, to maintain their individual immunological properties. The finalized vaccine construct comprised 347 amino acids (**Figure 1**).

### Population coverage analysis

**Figure 2:**
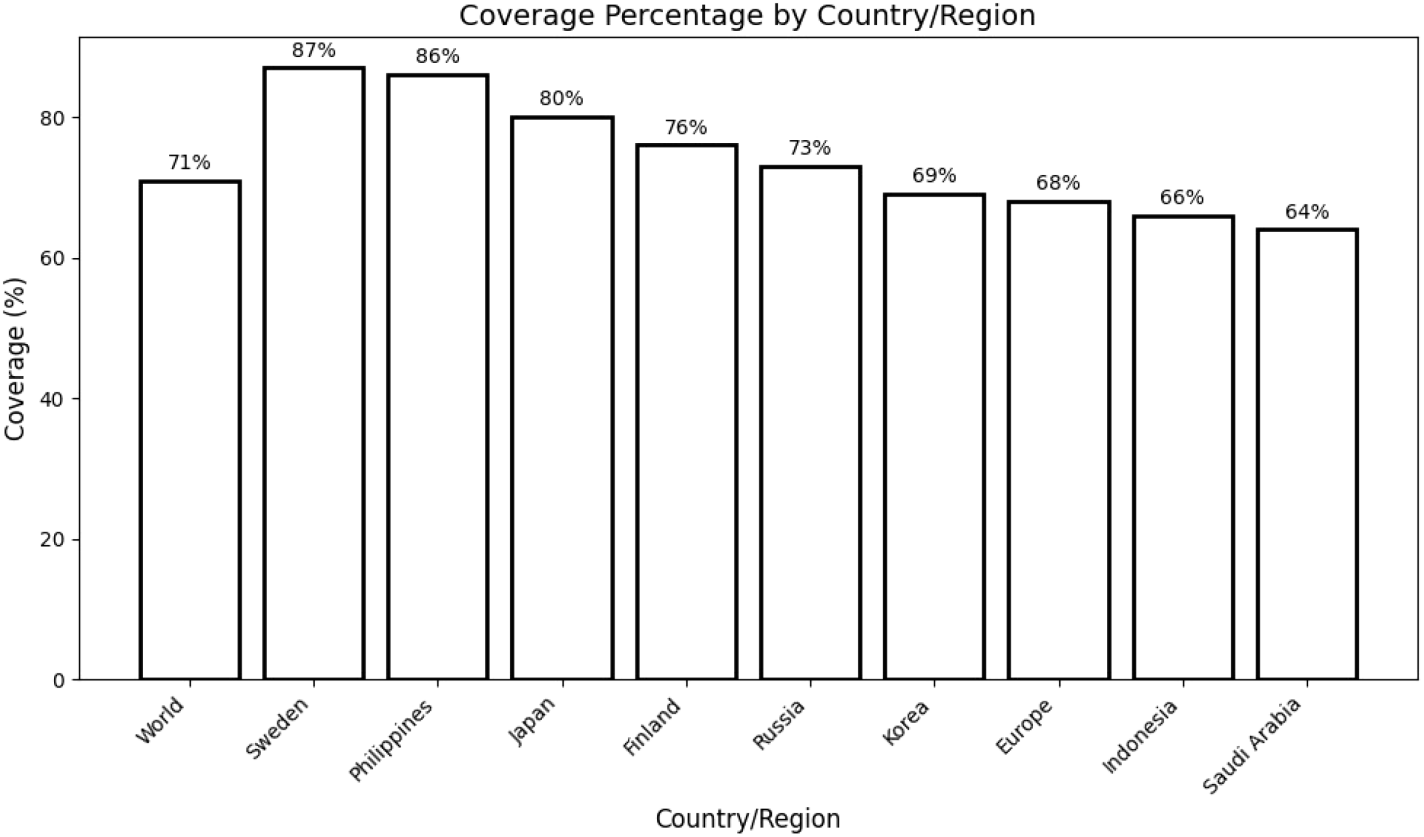
Population coverage analysis of selected T-cell epitopes across different countries/regions. The bar graph depicts the percentage coverage in the global population (71%) and specific regions, including Sweden (87%), the Philippines (86%), Japan (80%), Finland (76%), Russia (73%), Korea (69%), Europe (68%), Indonesia (66%), and Saudi Arabia (64%). This analysis highlights the broad applicability and potential impact of the designed multi-epitope vaccine in diverse populations.

A comprehensive population coverage analysis was performed on the selected CTL and HTL epitopes utilized in the development of the multi-epitope vaccine (MEV). The analysis revealed that the chosen epitopes collectively covered approximately 71% of the global population. Notably, the highest population coverage was observed in Sweden, with an impressive 87%. Other countries also exhibited significant coverage, including the Philippines (86%), Japan (80%), and Finland (76%). These findings substantiate the potential of the filtered epitopes as promising candidates for constructing an effective MEV targeting diverse populations globally (**Figure 2**).

### Post-analysis of vaccine structure

The stereochemical properties of the constructed vaccine were analyzed using the ProtParam tool. The vaccine structure exhibited a molecular weight of 38,154.87 Da and an isoelectric point (pI) of 10.30, indicating its basic nature. It contained 55 positively charged amino acids (arginine and lysine) and 23 negatively charged amino acids (glutamic acid and aspartic acid). The instability index of the structure was calculated as 27.14, classifying it as stable. Furthermore, an aliphatic index of 68.44 confirmed its thermostability, while the GRAVY (Grand Average of Hydropathicity) value of -0.464 indicated a hydrophilic nature. The half-life of the vaccine was predicted to be 30 hours in mammals (*in vivo*), over 20 hours in yeast (*in vivo*), and over 10 hours in *E. coli* (*in vivo*). Additionally, the vaccine was confirmed to be non-allergenic, non-toxic, and antigenic.

### Structural analysis of vaccine

**Figure 3:**
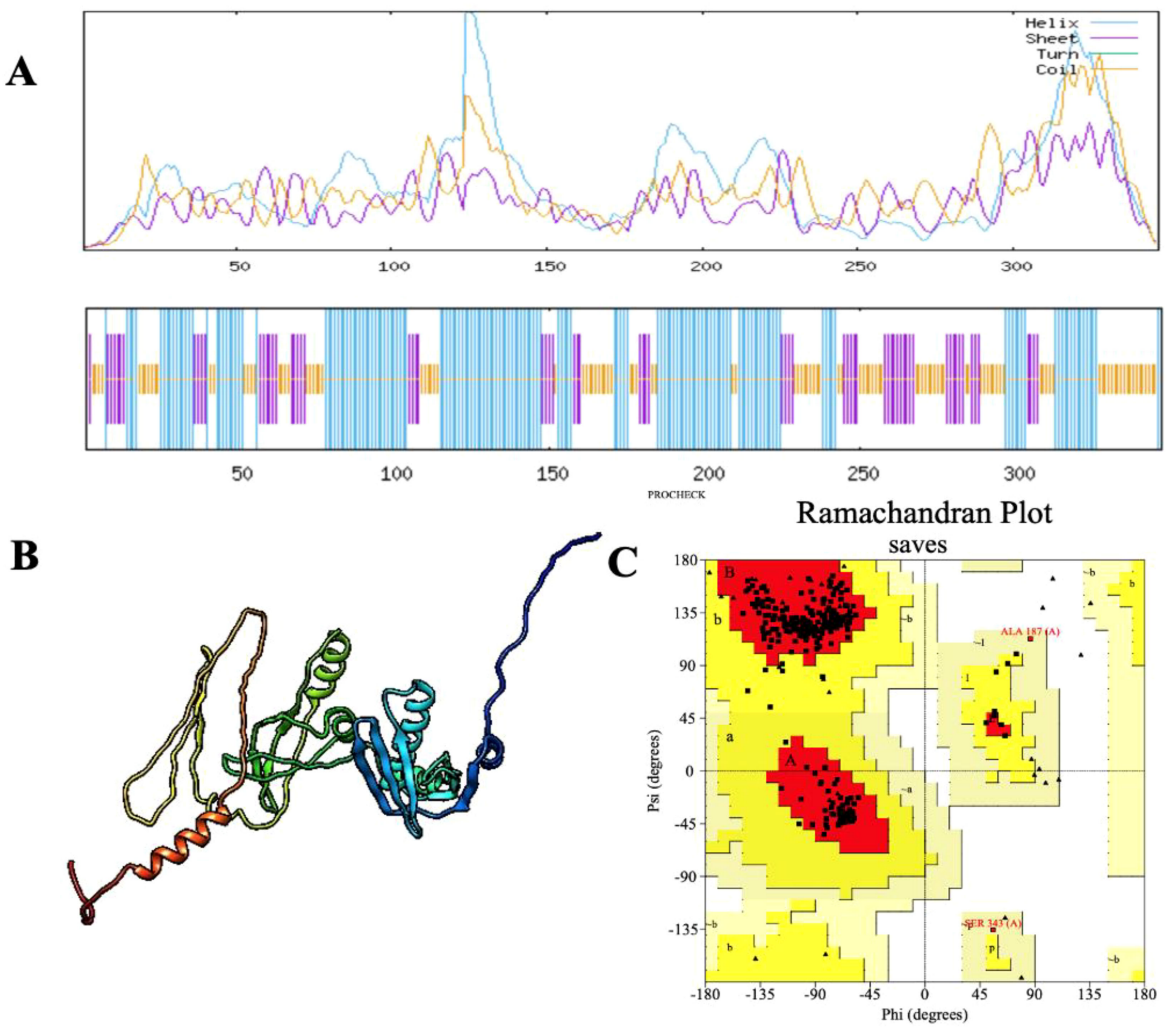
**(A)** Secondary structure prediction of the final multi-epitope vaccine construct using the SOPMA tool. The diagram illustrates the distribution of helices (blue), sheets (red), coils (purple), and turns (green). The horizontal black bar at the bottom represents the full length of the protein. **(B)** The refined 3D structure of the vaccine construct, displaying its spatial conformation. **(C)** Ramachandran plot analysis of the vaccine construct, demonstrating structural quality with 95.7% of amino acid residues positioned in favored regions.

**Figure 4:**
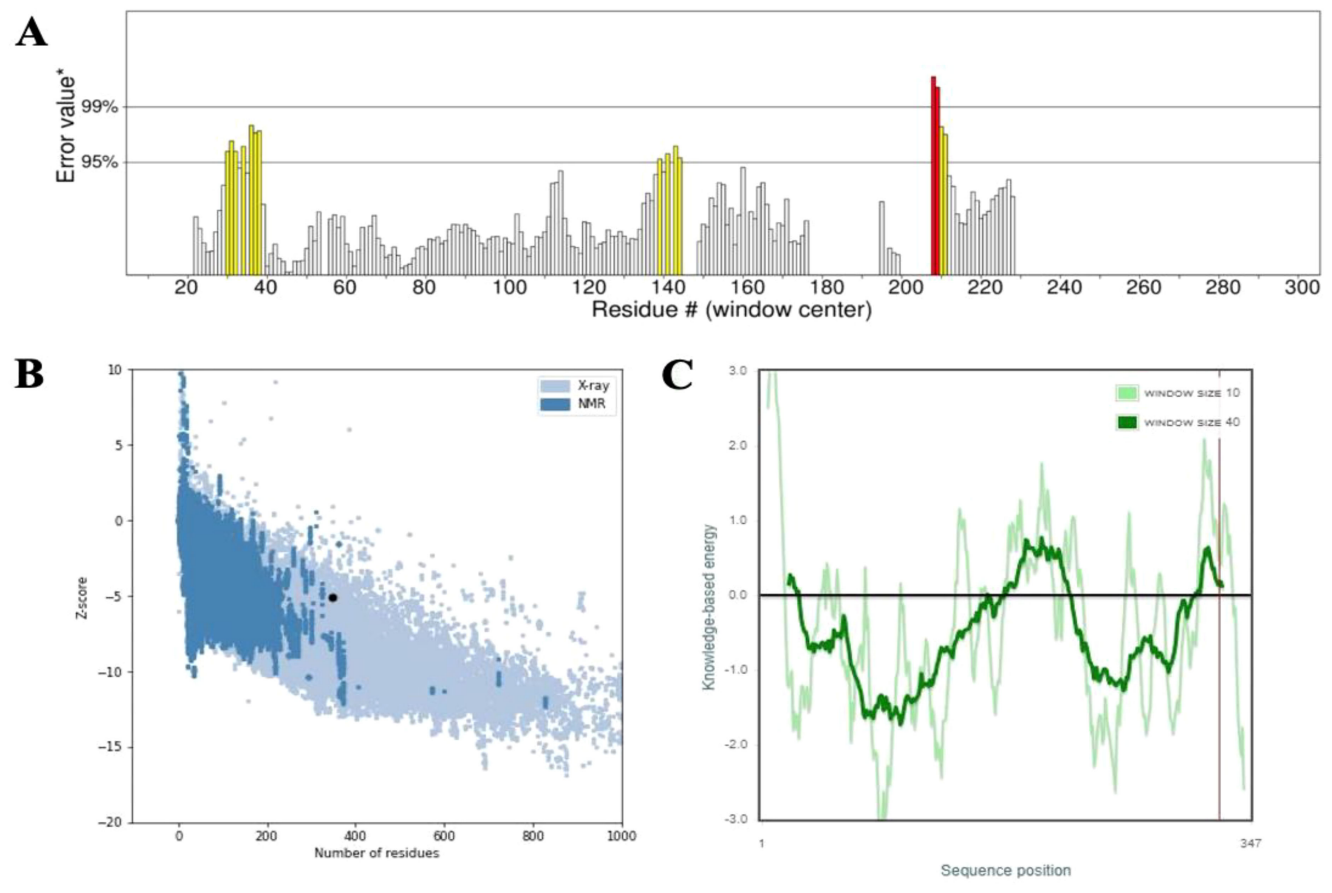
**(A)** Structural validation of the refined 3D vaccine model using the ERRAT tool. Regions of the structure rejected at the 99% confidence level are highlighted in red, while those rejected at the 95% confidence level are shown in yellow. **(B, C)** The Z-score plot of the refined 3D model, generated by ProSA-web, provides an assessment of the overall quality and reliability of the predicted vaccine structure.

Secondary structure analysis using SOPMA revealed that the 347-amino acid sequence comprises 162 residues forming α-helices (46.69%), 71 residues forming extended strands (20.46%), and 114 residues involved in random coils (32.85%), indicating a well-organized structural profile. The three-dimensional structure of the vaccine construct was predicted using the Alphafold server, followed by refinement through the Galaxy Refine server to optimize structural quality. Validation of the refined model was performed using a Ramachandran plot, which indicated that 95.7% of amino acid residues were located in the most favorable regions, 3.0% in the allowed regions, and 0.0% in the disallowed regions (**Figure 3**). Further evaluation demonstrated that the vaccine structure achieved a high-quality factor of 85.246 and a Z-score of -5.06, confirming the absence of poor rotamers (**Figure 4**).

### Selection of B-cell epitopes

**Figure 5:**
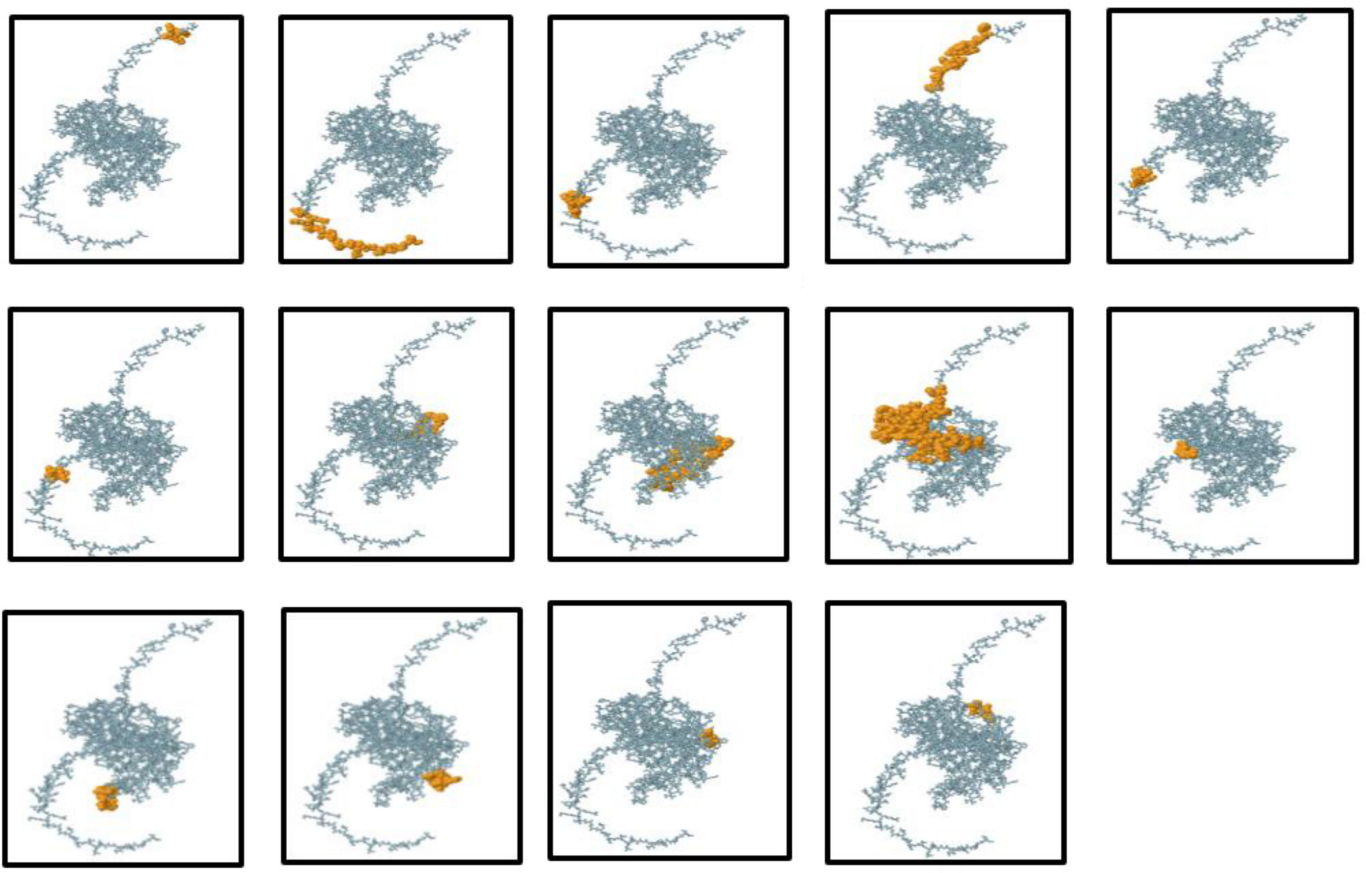
Three-dimensional representation of the conformational or discontinuous B-cell epitopes in the designed multi-epitope vaccine. The conformational B-cell epitopes are highlighted as an orange surface, while the remaining bulk of the polyprotein is depicted using grey stick representation.

B-lymphocytes play a pivotal role in humoral immunity by producing antibodies. Therefore, an effective vaccine must include optimal B-cell epitope domains to elicit a robust antibody response. In this study, 14 conformational B-cell epitopes, ranging from 3 to 53 residues in length, were identified with scores between 0.518 and 0.988. Additionally, 8 linear B-cell epitopes were predicted using the ElliPro server with default parameters. The conformational B-cell epitopes were visualized using PyMOL v1.3, a molecular graphics system, during the vaccine design process (**Figure 5**).

### Molecular docking with host immune receptor

**Figure 6:**
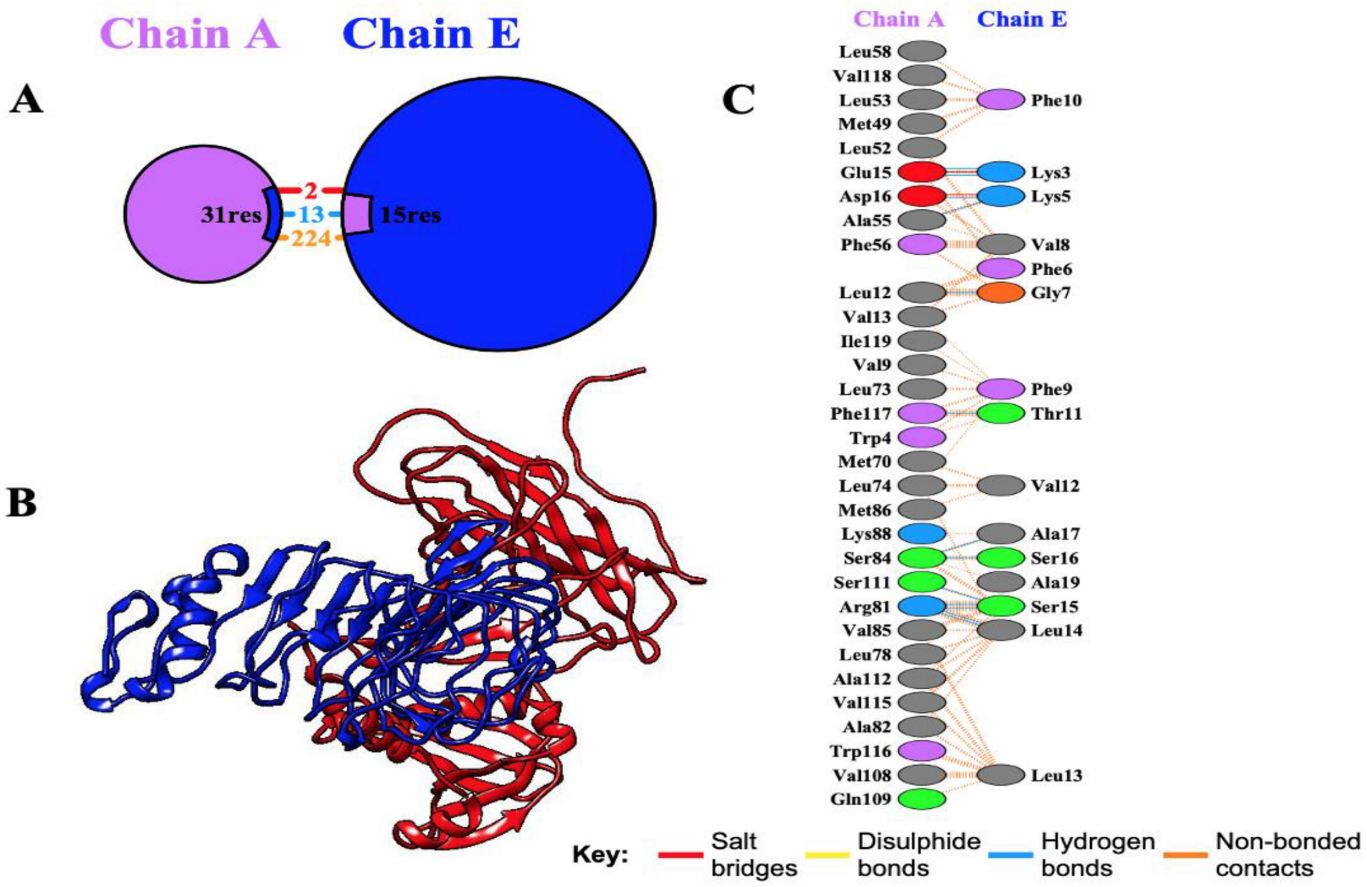
Docking analysis of the interaction between the receptor TLR4 and the multi-epitope vaccine (MEV). **(A)** Chain A and Chain E of the receptor and the MEV are depicted in purple and blue, respectively, highlighting the interacting residues. **(B)** Docking visualization showing Chain A of the receptor (blue) and the MEV (red), illustrating the optimal binding affinity. **(C)** Detailed illustration of interacting residues between the receptor and the vaccine construct, showing the formation of 13 hydrogen bonds between the receptor residues and the vaccine molecule.

Molecular docking is a critical technique for elucidating the binding interactions between vaccine constructs and immune receptor proteins. In this study, the molecular docking of the designed multi-epitope vaccine (MEV) with the human Toll-like receptor 4 (TLR4) was performed using the ClusPro server. ClusPro is a highly reliable protein-protein docking platform that integrates a hybrid docking algorithm, combining experimental substrate binding site data with small-angle X-ray scattering for docking analyses. The refined 3D structure of the vaccine construct (ligand) was docked against the TLR4 receptor (PDB ID: 3FE8), generating 10 docking models. The top-ranked docking model, with 230 members in its cluster and an interaction energy of -1277.0 kcal/mol, demonstrated high stability of the vaccine-TLR4 complex. Molecular interactions within the docking complex were analyzed using the PDBsum server, which revealed that the MEV construct exhibited favorable binding with chain A of the TLR4 receptor, forming 13 hydrogen bonds (**Figure 6**). Thermodynamic parameters for the binding energy of the docking complex were computed using the PRODIGY tool. The equilibrium dissociation constant (Kd) was determined to be 4.1×10−8 at 37°C, with a Gibbs free energy change (ΔG) of -10.1 kcal/mol. These results confirm the stability and strong binding affinity of the MEV construct to the TLR4 receptor.

### Normal mode analysis

**Figure 7:**
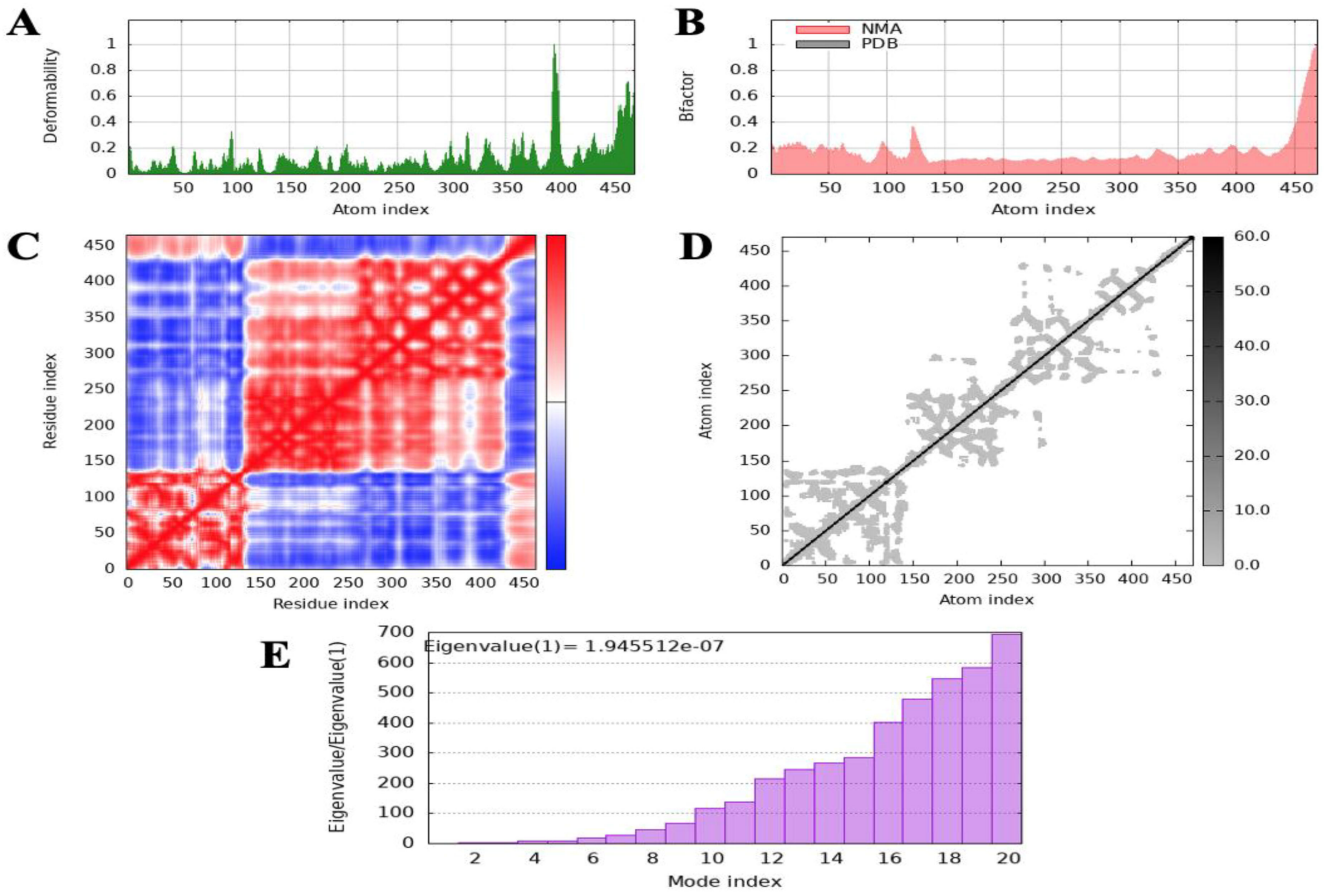
Molecular dynamics (MD) simulation analysis of the docked complex of the multi-epitope vaccine (MEV) with the receptor. **(A)** Deformability plot illustrating the flexibility of different regions in the docked complex. **(B)** B-factor analysis indicating the atomic fluctuations within the complex. **(C)** Covariance index depicting the correlated motions of residues. **(D)** Elastic network analysis demonstrating the connectivity and motion of residues within the complex. **(E)** Eigenvalue analysis representing the stiffness of the docked structure and its associated energy requirements.

Normal mode analysis (NMA) was performed to evaluate the molecular stability and functional motions of the MEV-TLR4 complex. The deformability plot revealed peak regions corresponding to main-chain residues exhibiting flexibility in the MEV-TLR4 complex. These highly deformable regions are indicative of “hinges” or “linkers” within the main chain. The experimental B-factor plot demonstrated the relationship between the NMA-predicted mobility and the MEV-TLR4 complex, showcasing the average RMSD values of the docked complex. The computed eigenvalue of the complex was 1.945512×10−7, reflecting the stiffness associated with each normal mode of motion. The variance bar illustrated individual (purple) and cumulative (green) contributions of each normal mode, indicating a negative correlation between variance and eigenvalue. Furthermore, a covariance map was generated to depict interatomic motions within the MEV-TLR4 complex. The map identified correlated (red), uncorrelated (white), and anti-correlated (blue) motions between different residue pairs. Additionally, a specialized elastic network model was constructed, representing the interatomic connections within the complex. The spring-like assembly between corresponding atoms and their stiffness were indicated by colored dots, with darker greys signifying more rigid interactions. Collectively, the NMA results demonstrated stable interactions and coordinated motions within the MEV-TLR4 complex, supporting its structural integrity and functionality (**Figure 7**).

### Immune simulations

**Figure 8:**
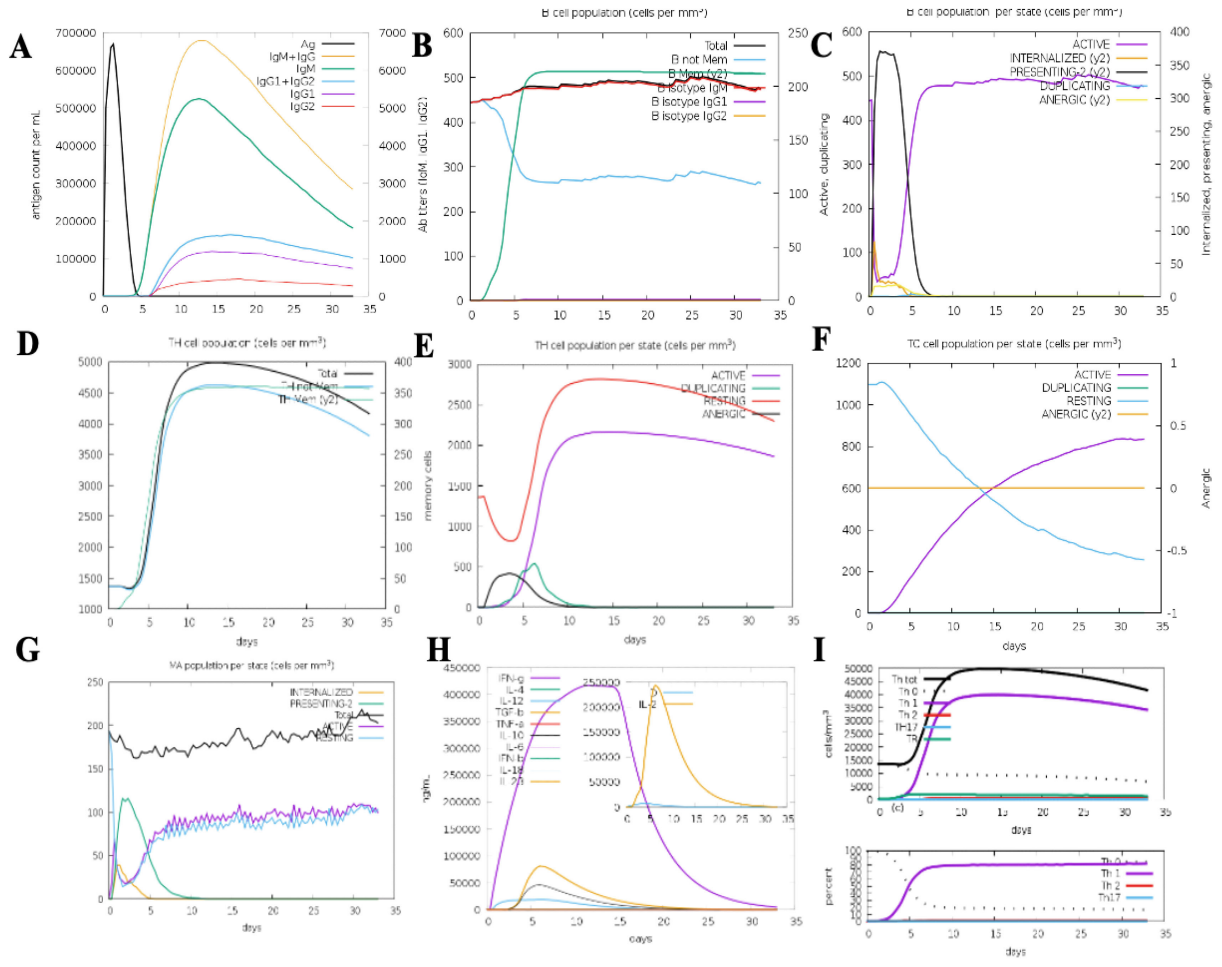
C-ImmSim immunization simulation results for the multi-epitope vaccine construct: **(A)** Immunoglobulin production depicted through color-coded peaks. **(B)** B-cell population showing increased types and class-switching potential. **(C)** Population distribution per state of B cells. **(D)** Evolution of T-helper cells over time. **(E)** Population distribution per state of T-helper cells. **(F)** Generation and dynamics of cytotoxic T cells. **(G)** Macrophage population distribution per state. **(H)** Cytokine and interleukin induction, showing elevated levels of IFN-γ and IL-2 post-vaccination. **(I)** Th1-mediated immune response activation.

The immune simulation results demonstrated a robust enhancement of both primary and secondary immune responses to the top-ranked vaccine construct. Administration of the vaccine led to elevated levels of immunoglobulins, including IgG1 + IgG2, IgM, and IgM + IgG, indicative of a strong antibody-mediated immune response. The B-cell population showed significant expansion upon repeated exposure to the vaccine antigen, highlighting the formation of humoral immune memory. The simulations also revealed a marked increase in cytotoxic T cells (CTLs) and helper T cells (HTLs), coupled with a substantial reduction in antigen levels during secondary and tertiary immune responses, underscoring the vaccine’s ability to enhance adaptive immunity. Additionally, the proliferation of natural killer cells, dendritic cells, and macrophages was predicted following each immunization cycle, reinforcing the construct’s capacity to stimulate innate immune responses. The vaccine also elicited cytokine and interleukin release, particularly IFN-γ, TGF-β, IL-23, IL-10, and IFN-β, which are crucial for mounting an effective immune response against infection. Notably, continuous antigen exposure during the immunization period resulted in significantly elevated levels of IFN-γ and TGF-β, while other cytokines were detected at lower concentrations. The calculated Simpson’s Index (D) confirmed a balanced immune response, reflecting the construct’s comprehensive impact on immune diversity. These findings suggest that the proposed vaccine construct can effectively activate T and B lymphocytes, inducing robust antibody production and establishing long-lasting memory cells upon repeated antigen exposure. The immune simulation results further support the potential of the vaccine construct to elicit strong innate and adaptive immune responses, demonstrating its efficacy in combating leishmaniasis (**Figure 8**).

### Codon optimization and *in silico* restriction cloning

**Figure 9:**
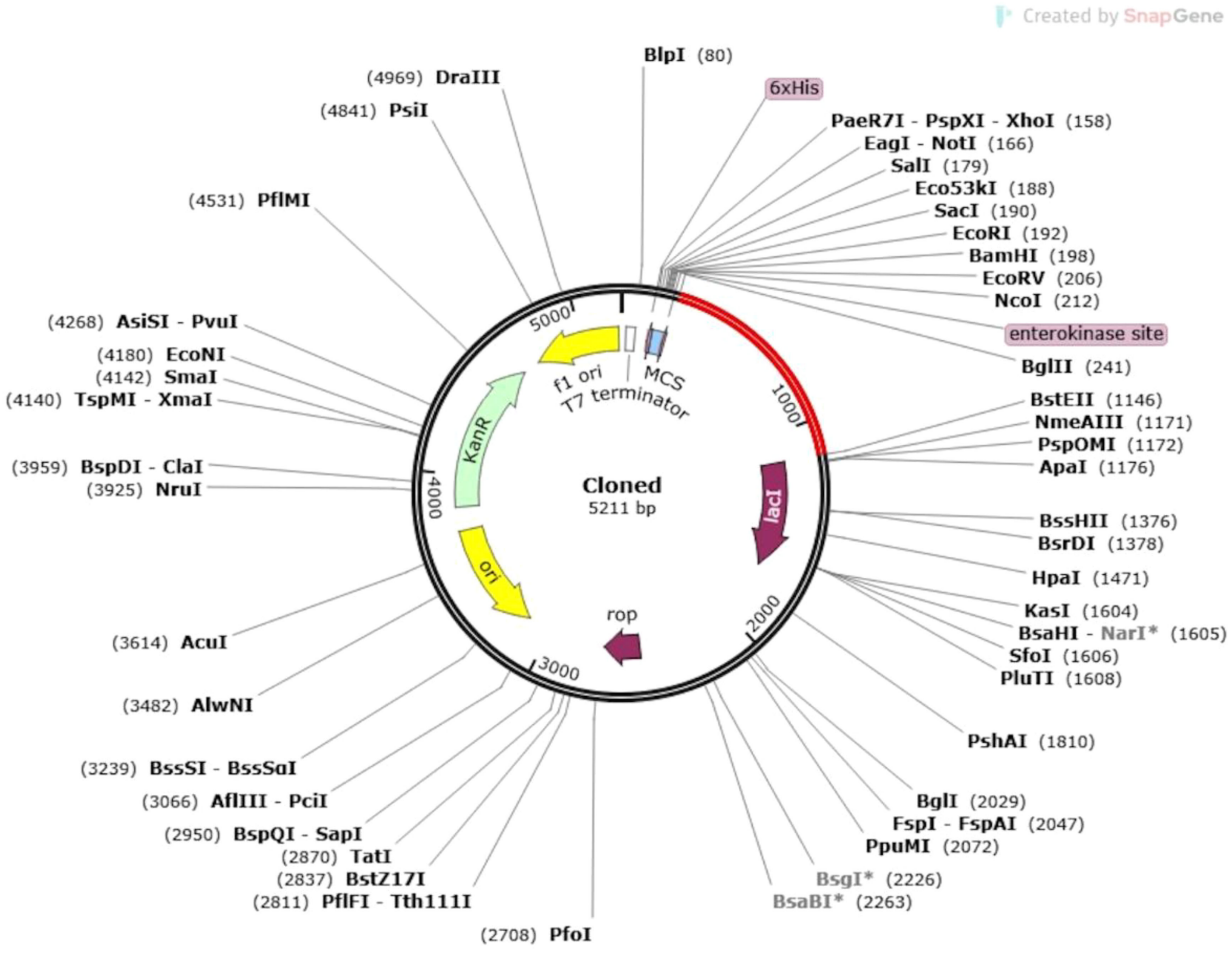
In silico cloning of the vaccine construct into the *E. coli* K12 host expression system. The plasmid backbone is represented in black, while the inserted nucleotide sequence of the vaccine construct is highlighted in red.

The expression potential of the proposed vaccine constructs was evaluated through codon optimization. Results obtained from the JCAT server revealed that all vaccine constructs achieved a Codon Adaptation Index (CAI) value of 1.0, indicating optimal codon usage. Furthermore, the GC content of the optimized cDNA sequences was 48%, which lies within the ideal range for efficient expression in the *E. coli* K12 vector. The optimized gene sequence of the prioritized vaccine construct was successfully integrated into the widely utilized pET30a(+) plasmid vector through *in silico* cloning. The total length of the recombinant plasmid was determined to be 5211 bp, confirming the feasibility of the construct for downstream applications (**Figure 9**).

## Discussion

*R. gnavus* is an important member of the human gut microbiota that plays both commensal and pathogenic roles (72). The involvement of this bacterium in diseases like inflammatory bowel disease (IBD) highlights its clinical significance, positioning it as a potential target for therapeutic interventions (4). Conventional treatments are challenging to implement because the bacterium is resilient and can evade immune responses, making the development of innovative solutions such as vaccines an urgent necessity (73). Vaccination remains one of the most effective strategies for reducing morbidity and mortality associated with microbial infections, particularly against emerging pathogens (74). Advances in immunoinformatics and reverse vaccinology offer a modern, cost-effective framework for rapid vaccine development, overcoming the limitations of traditional methods (75). These methodologies have successfully applied to propose vaccines for pathogens as diverse as *Mycoplasma pneumoniae*, *Salmonella Typhimurium*, and *Campylobacter jejuni*, among others (76, 77).

Here, a multi-epitope vaccine (MEV) construct against R. gnavus was designed using subtractive proteomics combined with immunoinformatics, molecular docking, and simulation techniques. Core proteome analysis identified essential proteins that are non-homologous to human proteins while exhibiting antigenic properties (42, 78). Such core proteins are crucial because they give the host a broad-spectrum protection against various strains of the pathogen (76). Among the identified proteins, the single-stranded DNA-binding protein (SSB) and FtsE are crucial for bacterial survival and virulence (74, 79). SSB is essential for maintaining genomic stability during DNA replication and repair, particularly under stress conditions, ensuring the resilience of *R. gnavus* (80). Its conservation across bacterial species underscores its importance in safeguarding replication fidelity, which can contribute to the persistence of *R. gnavus* in the gut, even during inflammatory states such as IBD (81, 82). Similarly, FtsE, a component of the FtsEX complex, is integral to bacterial cell division and peptidoglycan remodeling (83). In *R. gnavus*, FtsE likely supports robust cell wall integrity, enhancing survival and adaptability in competitive gut environments (83, 84). These proteins underscore the bacterium’s ability to endure host defenses and environmental stresses, making them potential targets for future therapeutic interventions.

Strict selection criteria were applied to identify CTL, HTL, and B-cell epitopes with high antigenicity while ensuring they were non-allergenic and non-toxic for potential use (85). Of extreme importance, the epitopes showed a very good worldwide population coverage, meaning an important potential for inducing immunity across different populations (28).

To increase the immunogenicity and stability of the vaccine, various linkers like AAY, KK, and GPGPG were used for joining the epitopes (86). These linkers have been reported to facilitate effective epitope processing, minimize junctional immunogenicity, and stimulate a robust immune response (87). The use of adjuvant cholera toxin subunit B coupled with the EAAAK linker ensured further stimulation of the immunity. This concept is very similar to a previous studies where these pairs were designed to improve stability and antigenicity of vaccines (76, 77).

Structural analysis of the vaccine construct showed that it was nontoxic, non-allergenic, and antigenic (75). Solubility predictions indicated that the vaccine would be easily expressible and bioavailable in the host system. This is important because solubility plays a crucial role in determining the effectiveness of subunit vaccines in producing strong immune responses (38). Docking studies showed strong binding interactions between the vaccine construct and the TLR4 receptor, which is a central component of the innate immune system (15, 64). Molecular dynamics simulations further confirmed that the vaccine-TLR4 complex is stable, highlighting its potential to mediate innate immune responses (42, 63, 64).

Codon optimization enabled the construct to be used for the expression of the vaccine in *E. coli* K12, with a codon adaptation index of 1.0 and a GC content of 48%, both indicating high efficiency of transcription and translation (69, 70, 76). Predictions from the immune simulation showed that it would trigger strong cellular and humoral immunity, which would also include strong T cell and B cell activation and the formation of memory cells, thus implying that the vaccine would offer long-term immunity against diseases caused by *R. gnavus*.

Although the findings are encouraging, this research has limitations. Predictions based on immunoinformatics are highly dependent on computational algorithms, which could not perfectly mimic biological outcomes. Hence, *in vitro* and *in vivo* studies that experimentally validate the safety and efficacy of the vaccine are needed. Further information on *R. gnavus* pathogenesis and host immune system interaction could narrow down vaccine targets and produce better outcomes. This is a rationally designed construct of a multi-epitope vaccine that would potentially activate robust immune responses against *R. gnavus*. Further experimental validations are required, but such a vaccine would be ideal for overcoming the challenges related to this opportunistic pathogen while maintaining gut microbiota balance.

## Conclusion

This study applied subtractive proteomics and reverse vaccinology to find vaccine candidates and design a multi-epitope vaccine against *R. gnavus* strain RJX1120. Pathogenic strain-specific antigenic proteins were selected to minimize off-target effects on beneficial gut microbiota. The identified antigens included Single-stranded DNA-binding protein and Cell division ATP-binding protein FtsE, promising as vaccine candidates. Epitopes predicted for B and T cells would generate both humoral and cell-mediated immunity. Adjuvants and linkers have been incorporated to increase their immunogenicity and stability. The proposed vaccine showed favorable structural and physicochemical properties, including strong binding affinity with TLR4 receptors, confirmed by molecular docking and simulation studies. Immune simulations predicted robust *in vivo* immunogenicity. Codon optimization and reverse translation ensured efficient expression in *E. coli.* Experimental validation in animal models is essential to confirm the efficacy and safety of the designed vaccine.

## Data Availability

The datasets presented in this study can be found in online repositories. The names of the repository/repositories and accession number(s) can be found in the article/supplementary material.
